# Exploring the Role of Rhodtestolin, A Cardio-Inhibitor from the Testes of *Rhodnius prolixus*, in Relation to the Structure and Function of Reproductive Organs in Insect Vectors of Chagas Disease

**DOI:** 10.3390/insects4040593

**Published:** 2013-10-30

**Authors:** Ralem Gary Chiang, Jennifer Ann Chiang, Hugh Hoogendoorn, Marli Maria Lima

**Affiliations:** 1 Biology Department, Redeemer University College, Ancaster, Ontario, L9K 1J4, Canada; E-Mail: jchiang@redeemer.ca; 2 Affinity Biologicals Inc., Ancaster, Ontario, L9G 4V5, Canada; E-Mail: hugh@affinitybiologicals.com; 3 Laboratorio de Ecoepidemiologia da Doenca de Chagas, Instituto Oswaldo Cruz-Fiocruz, Rio de Janeiro, RJ, Brazil; E-Mail: mmlim@ioc.fiocruz.br

**Keywords:** Reduviidae, Triatominae, *Rhodnius*, cardio-inhibitor, rhodtestolin, testes extracts, spermatophore, insect reproduction, Chagas disease

## Abstract

Rhodtestolin is a cardio-inhibitor that was first discovered in testes extracts of the blood-feeding insect, *Rhodnius prolixus*. Its role in reproduction remains unconfirmed, but if delivered to the female during spermatophore formation, it may serve to calm the female and/or relax the vaginal muscles to facilitate delivery and storage of the spermatophore. We describe here the anatomy of reproductive organs in *R. prolixus* and show that rhodtestolin is present in a low-molecular weight fraction of testes extracts separated by gel filtration, as well as in spermatophores delivered to the female during spermatophore formation. We also report that a rhodtestolin-like factor is present in the testes of *R. brethesi*, *Triatoma dimidiata*, *T. klugi* and *Nesotriatoma bruneri*, other Reduviidae, which are vectors of Chagas disease. Male secretions in insects are known to modify female behavior after copulation, and the presence of rhodtestolin in several genera of Reduviidae suggests that it plays an important role in reproductive success. Determining this role could lead to developing additional population control strategies for these bugs.

## 1. Introduction

In 2007, researchers from North and South America gathered together at the Seventh International Congress of Comparative Physiology and Biochemistry for a symposium on the research status of Reduviidae bugs, a group of blood-sucking cone noses that are vectors of Chagas disease [1]. This disease, also known as American trypanosomiasis, is caused by the flagellate parasite, *Trypanosoma cruzi*, which can be transmitted through the feces of the vector as it defecates on the host. The parasite infects muscle, and such infections lead to heart and/or digestive disorders. Chagas disease is one of 17 neglected tropical diseases with an estimated seven to eight million people infected with *T. cruzi* worldwide [2]. This symposium led to the present collaboration between Canadian and Brazilian institutions in which the study of a cardio-inhibitor, discovered in the testes of *R. prolixus* raised in North America [3], is being expanded to include the physiology of other Chagas disease vectors from Central and South America.

As predicted by Buxton in 1930 [4], the insect vector of Chagas disease, *R. prolixus*, has become one of the most popular insect models in which to study the digestion of blood and the physiology of blood-sucking insects. Its rise to popularity began with V.B. Wigglesworth who first studied this insect when working under Buxton at the London School of Hygiene and Tropical Medicine. Buxton’s laboratory colony originated from insects obtained around 1925 from Venezuela by the French parasitologist, E. Brumpt [5]. Years later, Wigglesworth was knighted for his contributions to insect physiology [6], and in much of his work he used *R. prolixus*. This insect was to become such a valuable experimental model that by the mid 1970s, colonies of this insect existed in laboratories around the world with nearly all of them originating from Brumpt’s original strains [5]. Indeed, the *R. prolixus* for the present study can be traced to the colony that Wigglesworth first encountered.

The convenience of *R. prolixus* as an insect model system ensured its continual use in teaching and research, which led to the discovery of the cardio-inhibitor associated with the testes. During a routine endocrinology teaching exercise, students found that only extracts from the testes caused the heart to go flaccid and stop beating. Subsequent studies showed that this effect was concentration dependent and could be reversed by removal of the extract [3]. We designated this factor as rhodtestolin (*Rhodnius* testes inhibitory factor), and its discovery has raised questions about its role in reproductive physiology. For instance, is it normally produced by the male to serve an important physiological purpose in the female? If so, could it be found in the spermatophore that is delivered to the female during copulation? Moreover, is a similar factor present in other insect vectors of Chagas disease, supporting the view that rhodtestolin may play an essential role in reproduction across species?

We report here the finding of a cardio-inhibitor in the spermatophore of *R. prolixus*, as well as in the testes of other triatomine vectors of Chagas disease: *Nesotriatoma bruneri*, *R. brethesi*, *Triatoma dimidiata*, and *T. klugi*. Rhodtestolin elutes within a single fraction from gel filtration indicating that it is likely a single molecule with the potential for purification and identification. These results support the hypothesis that rhodtestolin serves an essential purpose for reproductive success in these blood-feeding insects. Research into this factor promises to increase our understanding of the life history of these important vectors of disease.

## 2. Experimental Section

### 2.1. Insect Colonies

**Figure 1:**
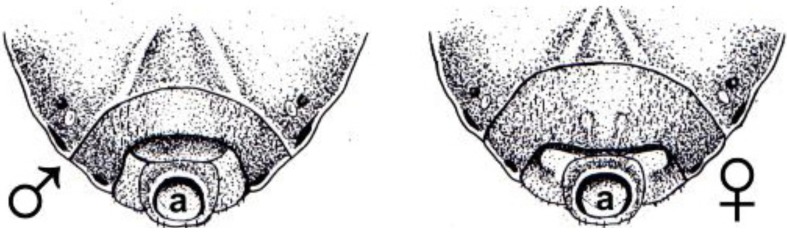
Line drawings illustrating the immature genitalia in L5 of *Rhodnius prolixus*. To distinguish the sexes, insects are placed ventral side up. With the use of a dissecting microscope the two concentric genital segments immediately around the anus (a) are examined. In the male (left diagram), the segment encircling the anus is entirely visible, not covered by the next anterior segment. Along its posterior edge, this next segment is approximately equidistant from the anus. In females (right side), the segment encircling the anus is partially covered at the midline by the next anterior segment, and there is little to no space between its middle posterior edge and the anus. Also, this segment in females often has a bilaterally symmetrical set of markings on either side of the midline, which is missing in the corresponding segment in males.

Adults of *R. prolixus* were taken from a colony maintained at Redeemer University College, and held in an insect growth chamber (Darwin Chambers Co., St. Louis, MO, USA) set at 28 °C, 80% humidity under a 12/12 hour light/dark cycle. Insects were housed in 500 mL wide-mouth plastic jars sealed with a wire mesh. They were reared either on the shaved bellies of rabbits, or on defibrinated rabbit blood using an artificial feeding method [7]. To avoid unwanted mating, insects were sorted visually into males and females at the fifth larval stage with the aid of a dissecting microscope to distinguish the immature male or female genitalia (see Figure 1).

Adults of *Triatoma dimidiata*, *T. klugi*, *Rhodnius brethesi*, and *Nesotriatoma bruneri* were reared in colonies set up from field insects and maintained at the Laboratory of National and International Reference on Triatominae Taxonomy of the Institute of Oswaldo Cruz-Fiocruz, Rio de Janeiro, Brazil. This facility maintains humidity at 77 ± 5%, temperature at 28 ± 3 °C, and uses natural lighting rather than a timed light cycle. The year in which and location at which the field insects were obtained are listed in Table 1. Every two weeks, insects were fed on live mice, *Mus musculus*, following a protocol approved by the Animal Use and Care Committee at Oswaldo Cruz Foundation, license L-081/08. Insects were housed in glass jars sealed with nylon mesh held in place with elastic bands. 

**Table 1:** The geographical origin of the colonies from which representative male and female adults were obtained and the date the colony was initiated.

Species	Geographical origin	Colony initiated
*Rhodnius prolixus*	Venezuela	approx. 1925
*Nesotriatoma bruneri*	Cuba	October, 2000
*Triatoma kluge*	Brazil (Nova Petropolis, Rio Grande do Sul)	November, 2000
*Rhodnius brethesi*	Brazil (Rio Arac, a/Rio Negro, Amazonas)	May, 2004
*Triatoma dimidiate*	Central America	December, 2006

### 2.2. Dissections

To expose the abdomen in either male or female insects, the non-anesthetized insect was secured ventral side down in a dissecting dish by placing strips of modeling clay over the legs. The wings were removed, and the insect covered with *Rhodnius* saline consisting of 129.0 mmol/L NaCl, 8.6 mmol/L KCl, 2.0 mmol/L CaCl_2_·2H_2_O, 8.2 mmol/L MgCl_2_·6H_2_O, 34.0 mmol/L D-glucose and 15.0 mmol/L Tris-HCl at pH 7.4 [8]. Using a blade breaker to fashion a scalpel from a piece of a razor blade, an incision was made in the dorsal abdominal cuticle along both lateral edges and transversely between the thorax and the abdomen. The cuticle was then lifted and teased away from the insect leaving the underlying epidermis intact. The dorsal vessel is recognized by its blue-green coloration, and is found embedded in the dorsal epidermis. For bioassays, no further dissection was needed, as a 50 to 100 µL stream of test saline could be applied directly to the heart region of the dorsal vessel with the aid of an automatic pipette. Heartbeats were observed using a dissecting microscope, and the heart rate was recorded.

To collect extracts of male reproductive organs, the abdomen of the male was exposed by removing the dorsal cuticle as described above. Microscissors were used to cut the dorsal epidermis along its lateral edges thus freeing it to be removed from the animal. The esophagus was pulled posteriorly into the abdomen by grasping the anterior end of the stomach with fine forceps. The alimentary tract was cut at the esophagus to prevent ingested blood from spilling into the abdominal cavity, and the entire alimentary tract was pulled out of the abdomen leaving the reproductive system exposed. A testis is located on each side of the abdomen at the level of the second to fourth abdominal segments. Each testis was lifted from the abdomen and was cut free from adhering tracheae with microscissors. Testes were collected and stored at −5 °C in *Rhodnius* saline.

To collect spermatophores, the reproductive system of recently inseminated females was exposed by removing the dorsal epidermis and alimentary tract as described above for the male. The vagina is located at the posterior end of the reproductive tract and is attached anteriorly to a common oviduct which, in turn, attaches to the left and right oviducts. The tract was cut at the level of the common oviduct, and the vagina containing the spermatophore was pulled away from the body. As with the testes, spermatophores were collected and stored at −5 °C in *Rhodnius* saline.

To prepare extracts, the collected testes or spermatophores were placed into a 2 mL hand-held glass homogenizer and homogenized for 5 min. The homogenate was centrifuged for 10 min. at 2,000 g, and the supernatant collected. Dilutions were made by adding *Rhodnius* saline to the extract.

### 2.3. Spermatophores

Adult male and female insects were housed in separate jars until individuals were brought together for mating. To increase the likelihood of successful mating, males were fed at least 3 days before they were offered a virgin female. To observe copulation, three virgin adult males were placed into a 65 mL clear plastic wide-mouth vial containing one virgin female and a 2.5 by 5.0 cm strip of filter paper. Increasing the number of males in the jar increases the chances that the female will copulate. The vial was sealed with a snap-on plastic lid into which a breathing hole was cut. Insects were observed in the light at room temperature. The duration of copulation was recorded, and immediately after the pair separated, the female was dissected to remove the vagina and the spermatophore it contained.

### 2.4. Gel Filtration Chromatography

For gel filtration chromatography, extracts were passed through a BioLogic DuoFlow chromatography system with an AV7-3 injection valve, and a superose-12 resin HR-10/30 column. The column was equilibrated with *Rhodnius* saline, pH 7.4 at 0.5 mL/min at 22 °C, and a 0.5 mL sample was injected through a 500 µL sample loop. One milliliter fractions were collected and absorbency read at 280 and 320 nm corrected for Rayleigh light scatter. Each fraction could be tested several times in the heart bioassay by using an automatic pipette to apply 50 µL of solution onto an exposed heart. 

## 3. Results and Discussion

### 3.1. General Body Plan

**Figure 2:**
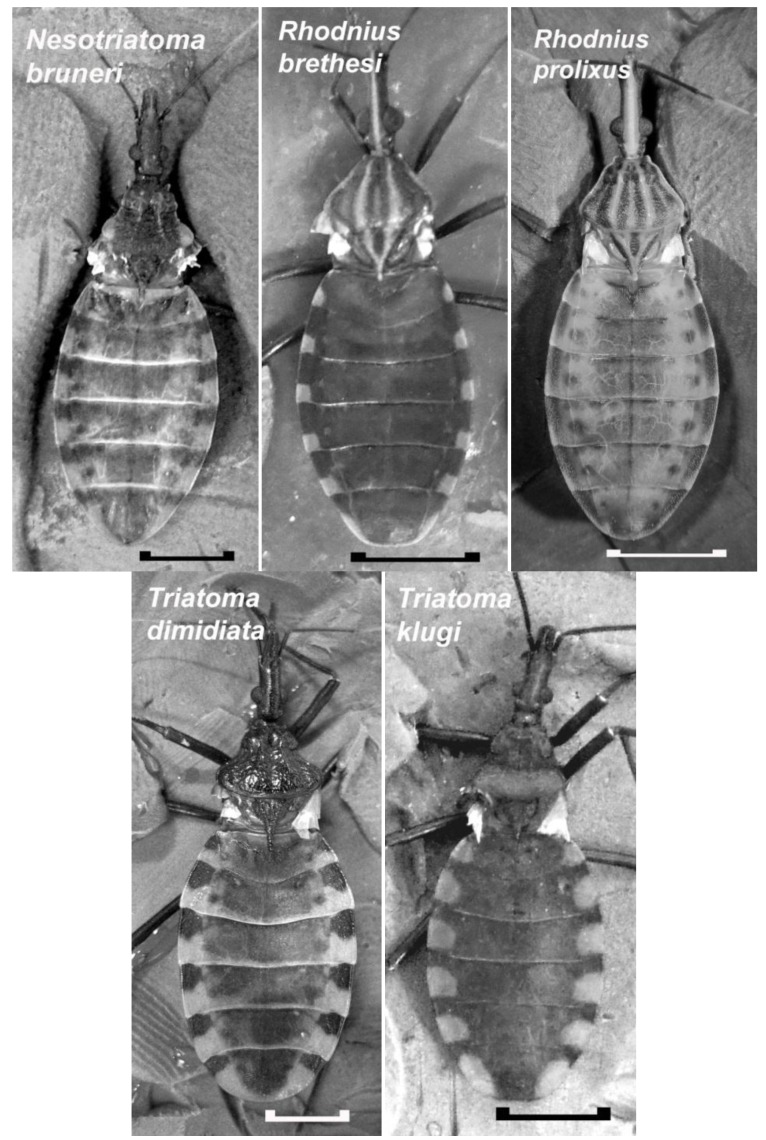
Dorsal view of the adult males of five species of Chagas disease vectors in which a cardio-inhibitor has been detected in crude extracts of the testes. Wings have been removed to show the similarity in the appearance of their dorsal abdominal cuticles. In these insects, the dorsal cuticle is mainly transparent and internal organs are visible when the lighting is adjusted to reduce glare. In these pictures, the dorsal vessel is clearly visible in *Neotriatoma bruneri* and *Rhodnius prolixus* as a dark line extending along the midline of the abdomen. It appears as a faint white line in *R. bresethi*. Scale bar: 5 mm.

Insect vectors of Chagas disease are opportunistic feeders that display a body plan designed for accommodating a large blood meal. As seen in the insects of this study (Figure 2), these insects have a characteristically large abdomen compared to the head and thorax. While they may vary in color (for color prints see [9,10]), the color patches are located in the same regions reflecting the similarity in the underlying tissue and muscle attachments. For the most part, the dorsal cuticle of the adult lacks pigmentation, especially towards the medial regions, and the internal organs can be visualized through the cuticle when the lighting has been adjusted to reduce glare. The ability to see through the cuticle of an intact insect was used previously to show that the heart rate of *R. prolixus* is inhibited by tactile stimulation of the sensory hairs along the ventral posterior side of the animal [8]. What cannot be seen from the dorsal aspect are the lateral abdominal folds in adults of *R. prolixus* and *R. brethesi*. These folds are adult features for *Rhodnius* and provide the surface area needed to allow the abdomen to stretch.

**Figure 3:**
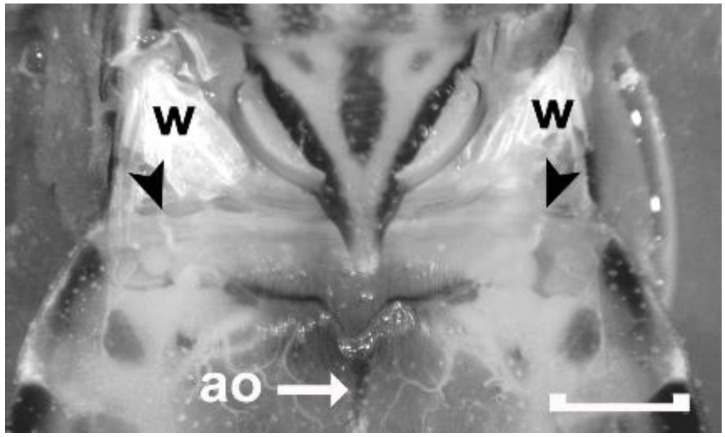
Dorsal view of the metathorax and the first full-sized abdominal segment (Segment 1–2) of an adult male *Rhodnius prolixus* covered with *Rhodnius* saline to increase the transparency of the cuticle. Wings have been removed leaving only their bases attached (w). Black arrowheads point to the first set of abdominal spiracles that are located on the dorsal side of the animal. The spiracles are not visible, but their corresponding tracheal trunks can be viewed through the transparent cuticle. The aorta of the dorsal vessel (ao) can also be seen through the cuticle. Scale bar: 1 mm.

Another morphological feature often overlooked in the abdominal cuticle of these insects is the first pair of abdominal spiracles. Unlike the rest of the abdominal spiracles, these are located on the dorsal side of the insect in a narrow flap of cuticle that is immediately in front of the first full-sized abdominal segment. These spiracles are not found along the lateral edge of the abdomen, but are positioned approximately one quarter of the distance to the midline (see Figure 3). The location of this first pair of spiracles identifies the first full-sized abdominal segment on the dorsal cuticle of these insects to be a fusion of abdominal Segments 1 and 2. Consistent numbering of the abdominal segments is important to ensure accuracy when comparing the work done on these insects by different researchers. For this study, and for comparisons to previous work, the abdominal segments are numbered Segment 1–2, Segment 3, Segment 4, *etc*. By applying this terminology, the most posterior full sized abdominal segment that houses the genitalia (not visible in the dorsal views in Figure 2) is abdominal Segment 7.

In addition to the abdomen being large, the structural components of both the nervous system and the circulatory system are arranged to permit these systems to continue to function when the abdomen becomes fully extended to accommodate the blood meal. The ventral nerve cord of many insects contains a ganglion in each of the thoracic and abdominal segments, but in Reduviidae, segmental ganglia in the abdomen are absent [11]. Rather than being located in the abdomen where they could experience considerable pressure from the expanded stomach, the abdominal segments are supplied by segmental nerves arising from the posterior ganglion located in the mesothorax. These abdominal nerves display some slack allowing them to be extended to a limited extent, and being located on the ventral side of the abdomen, these nerves are less subject to the stretch of the stomach. In adults, most stretch associated with feeding occurs in the dorsal cuticle.

Circulation is also maintained after engorging a blood meal because the alary muscles and ostia of the heart are located on the dorsal vessel to the rear of the abdomen at the level of the last two abdominal segments [12]. This region is not congested by the expanded stomach, and hemolymph is free to enter the heart which can propel a pulse of hemolymph into the aorta. The aorta stretches considerably along its length when the stomach expands, and it becomes flattened against the inner surface of the dorsal cuticle. Nevertheless, it maintains the ability to produce peristalsis which pushes the pulse of hemolymph up into the head, as can be visualized through the transparent cuticle. Although these insects appear unaffected if circulation is stopped, previous studies have shown that continued circulation to the head is important for long-term processes, such as egg production [13]. By localizing the heart to a region not congested by the engorged stomach, circulation can continue unimpeded.

### 3.2. Heartbeats

**Figure 4:**
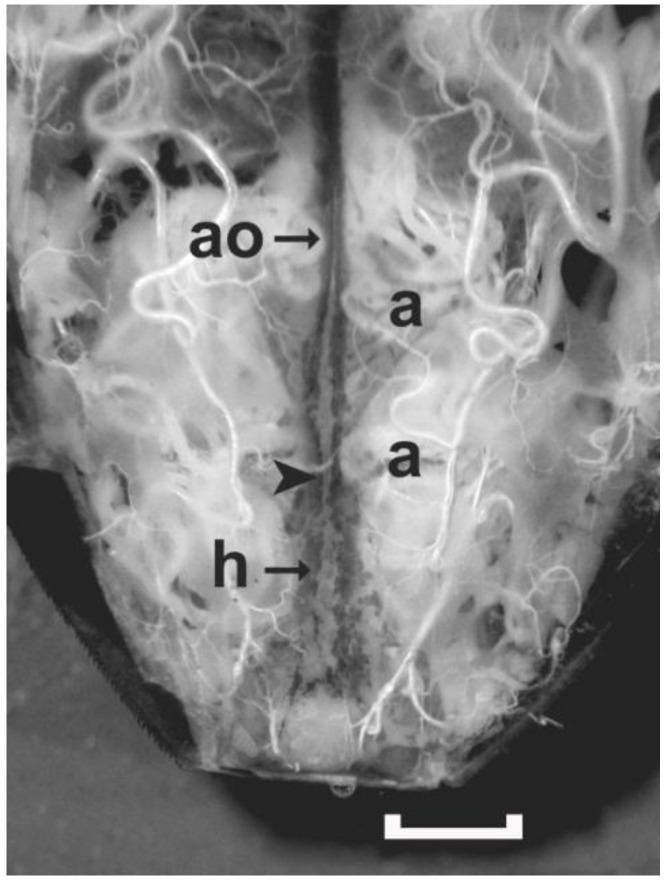
A live preparation covered with *Rhodnius* saline showing the posterior region of the dorsal vessel of an adult female *R. prolixus* exposed by removing the dorsal cuticle. When the alary muscles (two labeled as ‘a’) contract, the hemolymph enters the dorsal vessel through ostia located in the walls of the heart (h). The heart chamber contracts to propel hemolymph into the aorta (ao). The aorta initiates a peristaltic wave (at arrowhead), which constricts the lumen of the aorta pushing hemolymph up into the head. Only the dorsal alary muscles on the one side of abdominal Segments 6 and 7 are labeled. Scale bar measures 1 mm.

To carry out the heart bioassay, the dorsal vessel was exposed as shown in Figure 4. The heartbeat has been previously described in detail for *R. prolixus* [12], and the same beating pattern was observed in the other species examined here. In brief, a single beat in a spontaneously active heart begins with (1) alary muscle contractions which expand the heart chamber at the posterior end of the dorsal vessel allowing hemolymph to enter the heart, (2) constriction of the heart chamber by the heart muscles to push hemolymph into the aorta, and (3) peristaltic contraction of the aorta to push hemolymph anteriorly. Even though each action associated with the dorsal vessel can occur independently, all three actions are needed to ensure maximal flow of hemolymph. In a regularly beating heart, the heart rate can range from 1 bpm to close to 40 bpm. Rates above 40 bpm reduce circulation because the heart is not in diastole long enough for maximal filling. 

For determining the effect of a test solution on the heartbeat, a single constriction of the heart chamber that pushes hemolymph into the aorta was counted as a single beat. In some cases, the heart may appear to be actively beating as the alary muscles contract to expand the heart, and then relax to allow the heart to return to rest by elastic recoil. Unlike a contraction of the heart, this action does not constrict the heart chamber and no hemolymph is pumped forward into the aorta. In a regularly beating heart, a single contraction and relaxation of the alary muscles is usually followed by contraction of the heart. In irregularly beating hearts, the alary muscles may contract two or more times before a heartbeat occurs, and if counted as a beat, the heart rate would be overestimated.

### 3.3. Male Reproductive System

**Figure 5:**
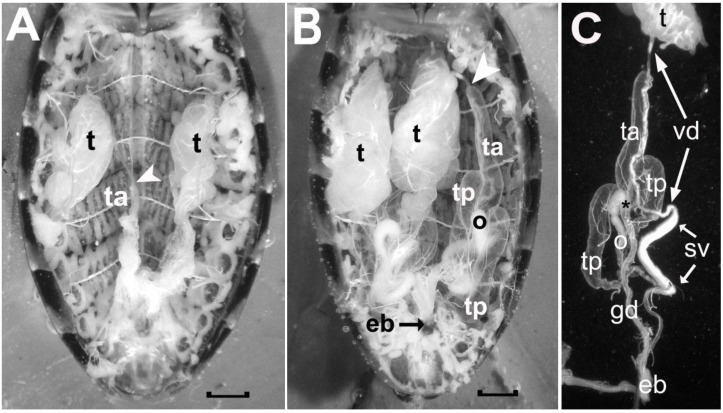
Photographs comparing the male reproductive system of a recently emerged adult male *R. prolixus* prior to ingesting a blood meal (A) and that of a two-month old adult male that has ingested more than one blood meal and has been mating (B). In C, the different components on one side of the reproductive tract of the older male are illustrated after these were teased apart. Symbols in A: t, testis; ta, empty anterior transparent gland; white arrowhead, location where the vas deferens from the testis attaches to the anterior tip of the anterior transparent gland. Symbols in B as in A with the addition of: ta, a filled anterior accessory transparent gland; tp, the two posterior accessory glands; o, opaque gland; eb, ejaculatory bulb. Symbols in C as in A and B with the addition of: gd, common duct of the accessory glands; vd, vas deferens; sv, seminal vesicle; asterisk, calyx of the glandular duct. Scale bars measure 1 mm.

The male reproductive system in Reduviidae is bilaterally symmetrical and each side consists of a testis and four accessory glands (see Figure 5). The testis is located laterally in the mid region of the abdomen and is supplied by trachea connected to tracheal trunks attached to Spiracles 3, 4 and 5 on the ipsilateral side. In recently emerged adult insects, the testes are well developed and extend between abdominal Segments 3 and 4 (Figure 5A). As they mature, they increase in girth and length and fill a large portion of the abdominal cavity from Segment 1–2 to Segment 5 (Figure 5B). The spermatozoa travel from the testis (t) down the vas deferens (vd) to be stored in the seminal vesicle (sv) before being delivered to the vagina by way of the ejaculatory bulb (eb) (refer to Figure 5C).

The accessory glands produce two morphologically distinct sets of secretions that the male delivers to the female after he has placed the semen into the vagina. During copulation, these two secretions are delivered one after the other [14]. The first secretion delivered after the semen is opaque in appearance, and is produced and stored in the opaque gland (‘o’ in Figure 5B and Figure 5C). The second secretion is transparent in appearance, and it constitutes the bulk of the spermatophore. This transparent material is made and stored in three transparent accessory glands. One transparent gland extends its blind end anteriorly (‘ta’ in Figure 5B and Figure 5C), and two extend posteriorly (‘tp’ in Figure 5B and Figure 5C). The bases of these glands converge at the calyx of the gland duct (asterisk in Figure 5C), and this common gland duct carries their secretions to the ejaculatory bulb. Unlike the testes, which are fully formed by the time of adult emergence, the accessory glands in recently emerged insects appear as small flattened sacs (see Figure 5A). Following a feed, they increase their girth and stretch longitudinally as they become filled with excretory material. In the older male (Figure 5B), the anterior transparent gland has stretched alongside its corresponding testis, and its anterior tip remains structurally associated with the vas deferens as indicated by comparing the white arrowhead in the younger insect to that in the older male. 

Our experience has shown that recently emerged unfed males do not mate, and to ensure that copulation occurs, adult males need to be fed a few days prior to mating. This observation, coupled with the appearance of the accessory glands in the unfed male, suggests that a lack of accessory gland material, and not the absence of spermatozoa, delays the mating behavior. When enough accessory gland material is available for mating, it is possible that a signal from the accessory glands to the nervous system provides the trigger for mating to begin. And it is possible that such a trigger could be carried by a hormone (an endocrine signal) or by the nervous system, because previous studies have shown that both mechanisms are available to the male. First, Sevala and Davey [15] found that the transparent accessory glands produce a polypeptide that is secreted into the body cavity. This substance could serve as a hormone. Second, Chiang and Davey [16] discovered a set of pressure receptors in the ventral body wall. These receptors are ideally situated to respond to pressure imposed by the enlarging accessory glands on either side of the insect.

**Figure 6:**
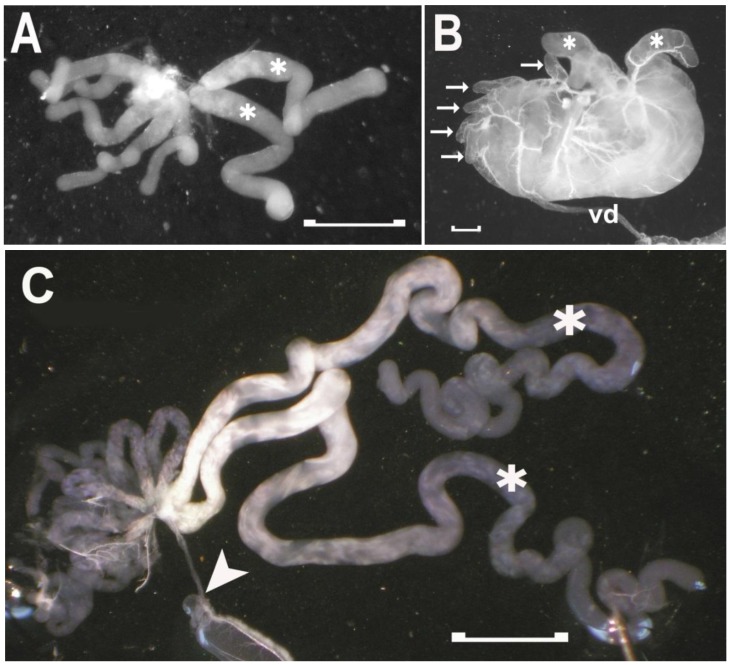
The testicular follicles and testis in *R. prolixus* taken from an L5 (A), a recently emerged adult (B), and a two-month old reproductively active adult (C). The five smaller follicles and two larger follicles (asterisks) are easily identified in the L5 (A). Even in an intact testis in B, the tips of the smaller follicles (arrows) can be distinguished from the two larger follicles. Testis growth with age is primarily due to growth of the two larger follicles. The larger follicles are approximately one and a half times longer than the smaller follicles in the young testis, but up to five to six times longer than the small follicles of the older testis (C). The vas deferens and the anterior transparent gland are not present in the L5, since these are adult structures that emerge in the process of metamorphosis. In C, the arrowhead denotes the attachment of the vas deferens to the transparent accessory gland. Scale bars: 0.2 mm.

Fully formed testes first appear in larval Stage 5 (see Figure 6). They do not undergo metamorphosis following feeding, but increase in size until the adult insect emerges. From the L5 stage to the older adults, the testes appear as a slightly flattened clump of tubules held together by a thin overlying membrane, which is associated with a fine mesh of tracheae. This membrane does not fully enclose the tubules of the testes, and their blind ends extend from the clump (see Figure 6B). These tubules are the testicular follicles, and in Reduviidae, each testis has seven follicles, which converge at their base to attach to the calyx of the vas deferens. Freitas *et al*. [17,18,19] have described the testicular follicles of several species of Reduviidae, and in all cases, two of the seven follicles are broader and several times longer than the other five follicles. We also observe this arrangement in *R. prolixus*, an arrangement, which is maintained from the L5 to the older actively mating male (see Figure 6). 

In the male involved in mating, the bulk of the testis consists of the two larger follicles, which contain material not visible in the smaller follicles. This morphological difference suggests different processes occurring in these two groups of follicles. The two larger testicular follicles may be involved in spermatogenesis and the production of spermatozoa, whereas the smaller testicular follicles could be involved in producing other secretions necessary for reproduction. Experiments are being planned to determine if the cardio-inhibitor from the testes is present in all the follicles or is localized in one of the two groups.

### 3.4. Testes Extracts

To determine whether a cardio-inhibitor discovered in *R. prolixus* is present in other Reduviidae bugs, we collected the testes from the species that were available at FIOCRUZ, and applied the testes extracts to the heart of the female of the same species. The presence of a cardio-inhibitor was detected when, upon application of a stream of testes extract over the heart, the heart immediately became flaccid, and beating stopped in diastole. In all cases, the application of a stream of control saline either had no effect on the heart rate or it increased its frequency for 30 seconds or more. The number of specimens of a species that were available for testing varied, and to avoid a false negative (*i.e.*, no effect due to the factor being too dilute in the extract) only those species having at least 10 fully mature well-fed adult males, a number that could provide 20 testes to prepare the extract, were considered to be the minimum number needed to suggest the absence of the factor. We observed cardio-inhibition in all the species that met this criterion. These species include *R. brethesi*, *Triatoma dimidiata*, *T. klugi* and *Nesotriatoma bruneri* (see Figure 2). The observation that this factor is found in triatomine bugs from different species and genera, and that structurally, the male reproductive system has been conserved across the species [10], suggests an important evolutionary role for this factor during sexual reproduction. Its role in evolution should become more apparent once its physiological role is determined. 

### 3.5. Cardio-Inhibitor in the Spermatophore

For rhodtestolin to have an effect on the female, it needs to be delivered during copulation as the male creates the spermatophore in the vagina. As noted above, the spermatophore is produced by the male first injecting semen into the vagina followed by a small amount of material from the opaque gland and then a larger amount of material from the transparent gland. The spermatophore is not immediately ejected after copulation, but remains in the vagina for several hours. This timing allows the spermatozoa to migrate to the spermatheca, a pair of blind-ended tubes, one attached to each side of the common oviduct [20]. Once a spermatophore casting is ejected from the vagina, the female can copulate again.

**Figure 7:**
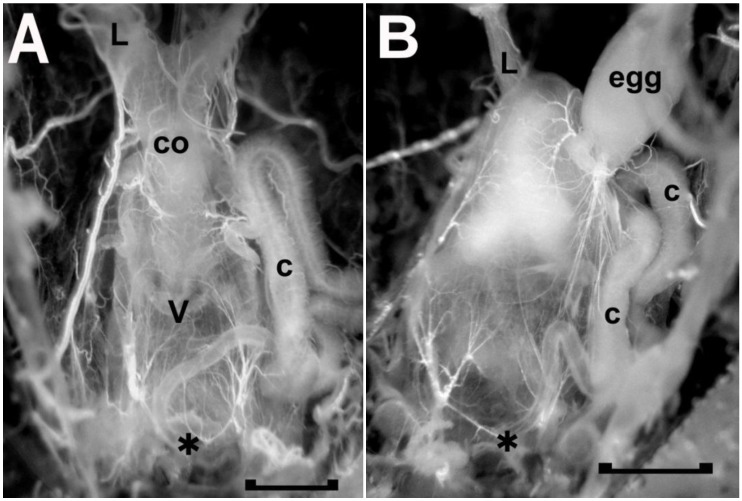
The freshly dissected female reproductive tract of two adult *Rhodnius prolixus*, one without (A) and one with (B) a spermatophore in the vagina (v). As seen in A, the anterior end of the vagina is attached to the common oviduct (co), which, in turn, leads to the two lateral oviducts (L, left lateral oviduct). The presence of a spermatophore, as seen in B, obscures the common oviduct as the spermatophore expands the vagina anteriorly. The spermatophore mainly consists of transparent material with opaque material at the anterior end next to the opening to the common oviduct. C, secretory portion of cement gland; asterisk, vulva and location where the duct of the cement gland secretes the cement onto the passing egg; egg, mature egg in base of right oviduct. Scale bar, 0.5 mm.

**Figure 8:**
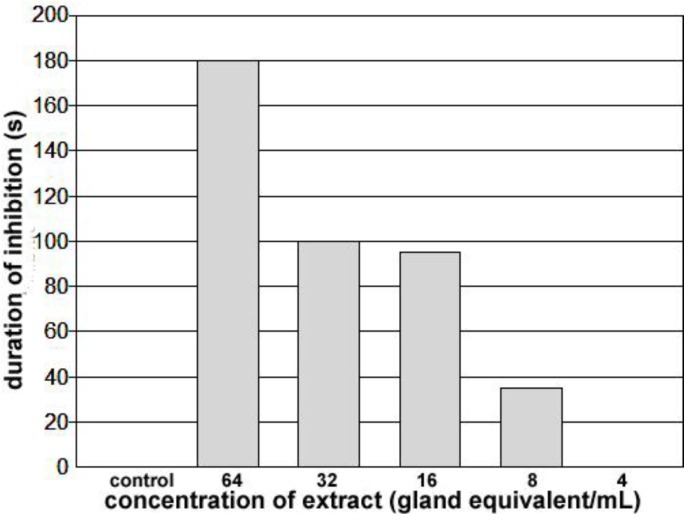
Duration of inhibition of the heartbeat for spermatophore extracts applied to a heart beating spontaneously at approximately 5 bpm for at least 10 minutes. Application of a 25–50 µL of control saline increased heart rate for approximately 30 s and application of less than 8 gland equivalents/mL did not slow the heartbeat. Application of concentrations of 8 or higher increased the duration of inhibition progressively. At 64 glands equivalents/mL, the heart remained quiet for greater than 3 minutes.

To remove spermatophores from recently copulating females, copulation was visually monitored. It lasted 52 ± 14 min (n = 26), and once the pairs separated, the female reproductive system was exposed and the vagina with the spermatophore removed. A spermataphore in the vagina can be seen through the walls of the vagina (Figure 7B). Being delivered first, the semen and opaque material are present at the anterior end of the vagina next to the common oviduct, and transparent material, delivered last, fills the bulk of this chamber. An extract was made from the spermatophores collected, and concentrations adjusted for testing. The bioassay was carried out on the same bug, and although the concentration effect needs to be verified with further tests, these results do establish the presence of a cardio-inhibitor in the spermatophore. The highest concentration tested (64 spermatophore equivalents/mL) inhibited the heart beyond the 3 min cut-off period, and inhibition was not observed below 8 spermatophore equivalents/mL (see Figure 8). As with the initial paper describing rhodtestolin [3], accessory gland extracts were also tested for the same species, but these extracts did not inhibit the heartbeat.

Assuming that the cardio-inhibitor plays an important role during sexual reproduction, a physiologically significant amount must be delivered in a single spermatophore. Although more tests need to be completed to verify the concentration effects described above, it is possible that only one spermatophore equivalent should be needed to inhibit the heartbeat if rhodtestolin’s role in sexual physiology is directly related to heartbeat inhibition. Our concentration effects therefore suggest that rhodtestolin may play a role that functions at lower concentrations. One such role would be to prevent the vaginal muscles from contracting since their contractions would expel the spermatophore from the vagina. The functional anatomy of vaginal muscles has been described previously [21], showing that they will contract in response to stretch. Since the spermatophore, which stretches the vagina, remains in position for several hours, any stretch reflex associated with the vaginal muscles must be delayed. The role of rhodtestolin on the contractions of the vaginal muscles is currently being investigated.

### 3.6. Gel Filtration of Rhodtestolin

**Figure 9:**
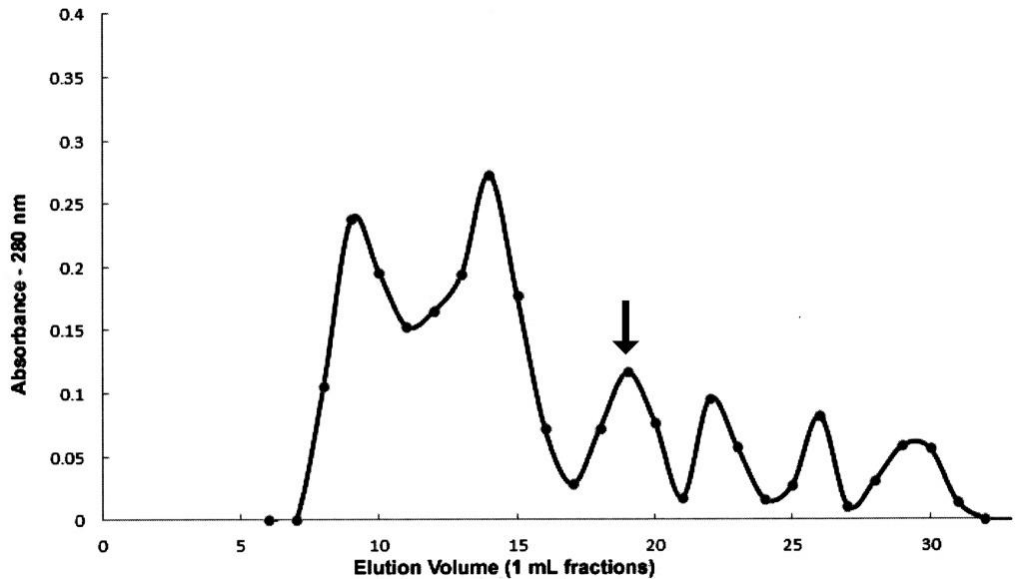
Gel filtration chromatography of the testes extract. Zero point five milliliters sample was applied to a Superose-12 (10/30) column with a bed volume of 23.6 mL. Fractions (1 mL) were collected at ambient temperature. Proteins elute in descending order of molecular weight, proteins of a larger mass elute early and smaller proteins elute later. Each fraction was tested by bioassay, with only Fraction 19 (arrow) exhibiting activity.

We are currently investigating several approaches to determining the chemical nature of rhodtestolin, and gel filtration chromatography has provided some interesting information. Gel filtration of the testes extract resolved six protein peaks as determined by absorbance at 280 nm (see Figure 9). Of these six peaks, only the third peak exhibited activity in the heart bioassay. Comparison of this elution profile to the elution profile of known molecular weight standards (Gel Filtration Standard, BioRad Laboratories Inc., Hercules, CA) run under the same conditions, showed that the position of the activity (2.5 void volumes) is consistent with a mass of about 5,000 daltons. Additional experiments are needed to further elucidate the nature of this activity.

## 4. Conclusions

A cardio-inhibitor is found in more than one species of insect vectors of Chagas disease, suggesting that it serves an important function in reproductive physiology. Because its effect is concentration dependent, those species in which it was not detected need to be re-examined when more specimens are available for study.

Rhodtestolin can be separated from the other proteins of the testes in a relatively pure form, because no other fraction, including those immediately before and after the active fraction, contained rhodtestolin activity, and the active fraction corresponds to a peak in absorbency. Rhodtestolin may prove to be a smaller molecule bound to a protein eluting in this fraction, but the ability to isolate it to a single fraction will facilitate future work designed to determine the chemical identity of this substance and its physiological activity on other insect tissues.

The presence of a cardio-inhibitor in the spermatophore extracts suggests that rhodtestolin enters the vagina during copulation. If inhibition of the heartbeat is due to it acting as a muscle relaxant, then rhodtestolin may relax the vaginal muscles, which, in turn, would allow the spermatophore to remain in the vagina for longer periods of time.

A previous study reports that the structure of the male reproductive glands is maintained across several species of Chagas disease vectors [10], and the present study suggests that male sexual physiology is also conserved because a cardio-inhibitor from the testes is common in five species from three different genera. Therefore control strategies focusing on rhodtestolin may be applicable to more than one species of Chagas disease vector, an important consideration since Chagas disease can be transmitted by several species of Reduviidae.
